# Durable Response to Glofitamab in HIV‐Positive Refractory Burkitt Lymphoma: Case Report and Discussion

**DOI:** 10.1002/jha2.70243

**Published:** 2026-02-12

**Authors:** Elisa Bozzotto, Theresa Magnes, Dominik Kiem, Barbara Zellinger, Daniel Neureiter, Arno Beer, Alexander Egle, Thomas Melchardt

**Affiliations:** ^1^ Department of Internal Medicine III With Hematology, Medical Oncology, Hemostaseology, Infectiology, and Rheumatology Paracelsus Medical University University Clinic Salzburg Salzburg Austria; ^2^ Institute of Pathology Paracelsus Medical University University Hospital Salzburg (SALK) Salzburg Austria; ^3^ Cancer Cluster Salzburg Salzburg Austria

**Keywords:** bispecific antibodies, Burkitt lymphoma, glofitamab, HIV, refractory lymphoma, time‐limited therapy

## Abstract

**Background:**

Refractory Burkitt lymphoma (BL) has a dismal prognosis after failure of frontline therapy. New treatment options are urgently needed.

**Methods:**

We describe a 60‐year‐old HIV‐positive patient with refractory and secondary primary BL after frontline and salvage therapy failure, treated off‐label with glofitamab successfully.

**Results:**

Twelve cycles of glofitamab were well tolerated and led to complete metabolic remission confirmed by imaging and biopsy, sustained for nine months post‐treatment.

**Conclusion:**

Glofitamab may represent a promising stand‐alone option in relapsed BL, when conventional cytotoxic therapies are exhausted.

**Clinical Trial Registration**: The authors have confirmed that clinical trial registration is not needed for this submission.

## 1 Introduction

Burkitt lymphoma (BL) is classified as a rare form of a mature B‐cell non‐Hodgkin lymphoma, which is characterized by significant aggressiveness. For adults, this type of lymphoma accounts for less than 5% of all non‐Hodgkin lymphomas [1]. Unlike children, adults usually present with more nodal, bulky disease [2]. Such a presentation is coupled with more clinical heterogeneity alongside comorbidities that complicate management.

First‐line treatment usually consists of short‐duration, high‐intensity combination chemotherapy and CD20‐targeted monoclonal antibodies. Fit adults are offered standard therapy with Cyclophosphamide, Vincristine (Oncovin), Doxorubicin (Adriamycin), and High‐dose Methotrexate (CODOX‐M)/Ifosfamide, Etoposide, and High‐dose Cytarabine (IVAC) or variants of the acute lymphoblastic leukemia regimen [3], which are well established. Rituximab, etoposide, prednisone, vincristine, cyclophosphamide, and doxorubicin (DA‐EPOCH‐R), however, is less toxic and is a viable option for patients without CNS involvement [4, 5]. While the initial response to therapy is often favorable, the prognosis in patients with refractory disease is poor without a universally accepted salvage treatment [6].

Autologous hematopoietic stem cell transplantation (auto‐HSCT) may offer benefit in patients who achieve chemosensitive disease; however, success with second‐line regimens in relapsed BL remains abysmal, and achieving sufficient disease control before transplantation is the major challenge.

Allogeneic HSCT (allo‐HSCT) may be considered in selected cases [7], but when remission is achieved, outcomes with auto‐HSCT appear at least comparable. Recent EBMT registry data presented at the ICML Lugano 2025 meeting (abstract) reported similar survival rates between auto‐ and allo‐HSCT among eligible patients, underscoring that pre‐transplant disease control remains the key prognostic factor [8].

Most centers reserve consolidation for patients responding to salvage treatment [9].

Emerging approaches, such as CAR‑T cell therapy and novel immuno‐oncology agents, are now being explored to address the significant unmet need. CAR‐T cell therapies, alone or combined with autologous transplantation, have been evaluated in relapsed BL [10]; however, outcomes remain unsatisfactory. Response is often short‐lived, and overall prognosis remains poor [11].

Small series and case reports suggest bispecific antibodies—like glofitamab (CD20 × CD3) and blinatumomab (CD19 × CD3) can induce meaningful remissions in heavily pretreated patients, enabling bridging to HSCT or maintenance in select cases. While early data are encouraging, they remain preliminary, underscoring the need for systematic evaluation within clinical trials [12, 13, 14, 15, 16, 17].

Glofitamab is a bispecific monoclonal antibody that targets CD20 on B cells and CD3 on T cells, redirecting T‐cell cytotoxicity toward malignant B cells. It has shown efficacy in relapsed or refractory diffuse large B‐cell lymphoma [15, 16] and early reports suggest similar potential in BL [14, 18]. Promising results have also been described in primary central nervous system lymphoma [17].

Here, we report a case of a 60‐year‐old HIV‐positive man with a refractory BL, who achieved remission after glofitamab therapy after failing two lines of salvage chemotherapy and being deemed ineligible for chimeric antigen receptor T‐cell therapy.

## 2 Materials and Methods

The patient was treated at the Department of Internal Medicine III (Hematology, Oncology, and Infectiology) at the University Clinic Salzburg. The patient was included in the Lymphoma Registry of the Austrian Group for Medical Oncology and provided written consent.

Histopathological evaluation was performed on formalin‐fixed, paraffin‐embedded lymph node biopsy specimens obtained at initial diagnosis in 2015 and at disease presentation in 2023. Immunohistochemistry was conducted according to institutional standards and included staining for CD20, CD10, BCL6, BCL2, c‐MYC, and Ki‐67.

The B‐cell clonality was assessed using the IdentiClone immunoglobulin heavy chain (IGH) Gene Clonality Assay (Invivoscribe) according to standardized BIOMED‐2 protocols. Genomic DNA was amplified via five multiplex polymerase chain reaction (PCR) master mixes targeting the conserved framework (FR1–3), diversity (DH), and joining (JH) regions of the immunoglobulin heavy chain gene. Amplicons were detected using the QIAxcel capillary electrophoresis system (Qiagen), with an analytical sensitivity of 5%. All reactions were validated with appropriate internal controls, including water, polyclonal control, and clonal controls (not shown).

### 2.1 DNA Extraction

DNA was automatically extracted from 2015 and 2023 BL blocks and 2023 normal tissue using a Maxwell RSC DNA FFPE Kit (Promega). Tumor samples were histomorphologically verified to contain 85%–95% tumor cells. Concentration was measured via Quantus fluorometry.

### 2.2 Mutational Analysis

NGS was performed using the Illumina Cancer HotSpot Panel on a MiniSeq platform (2 x 151 bp paired end). Data were analyzed with JSI SeqPilot (v4.4.0) against the GRCh37 (hg19) reference genome. Variants were called according to HGVS standards using a minimum 500× coverage and a 5% allele frequency threshold.

## 3 Results

The patient, a 53‐year‐old man at the initial presentation, was diagnosed with HIV infection in July 2015 and immediately started antiretroviral therapy (ART) with lamivudine (3TC), abacavir (ABC), and raltegravir (RAL). His viral load became undetectable soon after treatment initiation, with a single transient viral “blip” in December 2015.

Later in 2015, he was diagnosed with BL and achieved complete remission after treatment with the GMALL B‐NHL protocol.

ART was simplified in September 2016 to a single‐tablet regimen containing 3TC, ABC, and dolutegravir (DTG), and later adjusted in May 2023 to 3TC plus DTG. He received prophylaxis with trimethoprim‐sulfamethoxazole and azithromycin to prevent *Pneumocystis jirovecii* pneumonia, toxoplasmosis, and atypical mycobacterial infections, which were discontinued after CD4 recovery to >200/µL (January 2017) and >50/µL (April 2016), respectively. Despite sustained virologic suppression, CD4+ T‐cell counts remained low, rising slowly from a nadir of 12/µL.

He remained in remission until July 2023, when he re‐presented with widespread lymphadenopathy involving cervical, mediastinal, hilar, and inguinal nodes.

Comparative histopathological and molecular analyses were performed on lymphoma specimens obtained at initial diagnosis in 2015 and at disease presentation in 2023. Both samples demonstrated classic BL morphology and an identical immunophenotype, including expression of CD20, CD10, BCL6, and c‐MYC, absence of BCL2 expression, and a high proliferative index (Ki‐67 approximately 100%).

**FIGURE 1:**
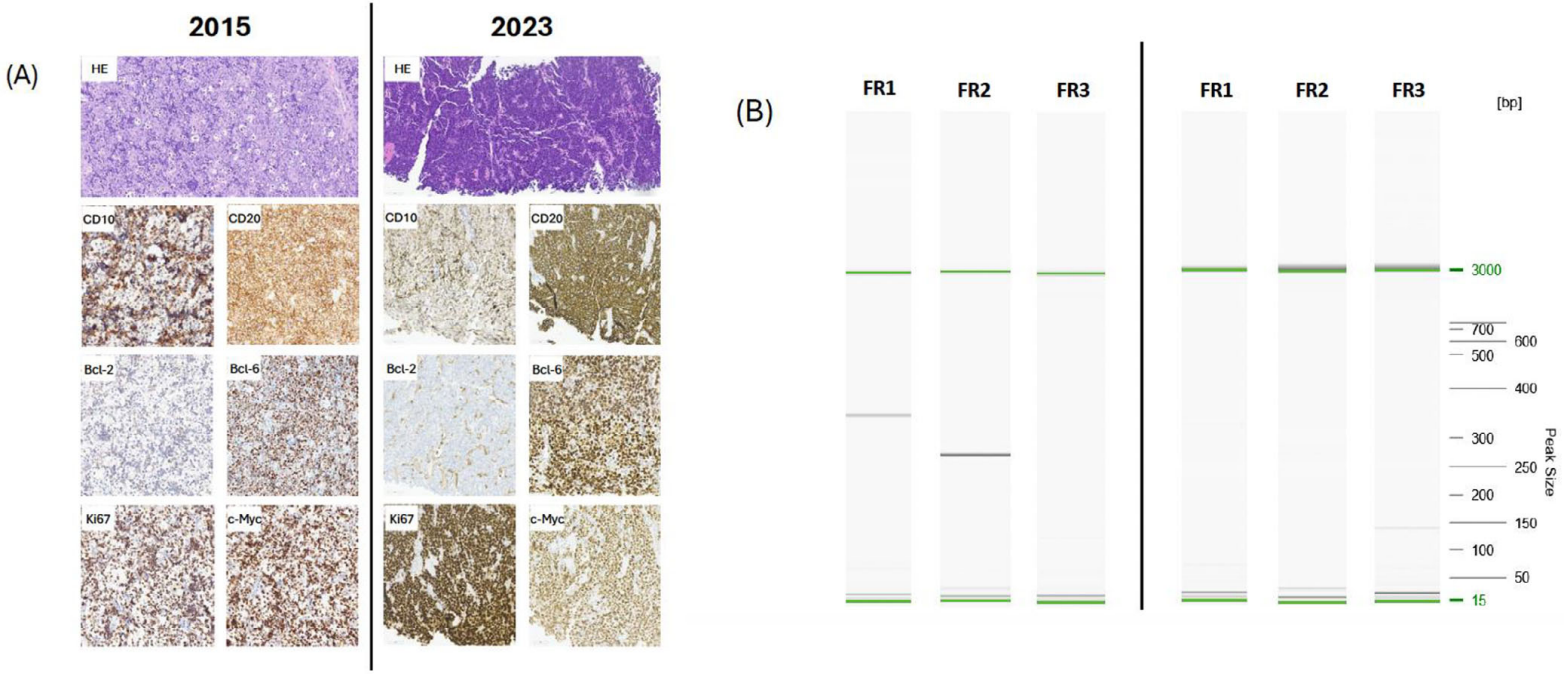
Immunohistochemical profiles and molecular clonality analysis of the case report with Burkitt lymphoma diagnosed in 2015 (left) and 2023 (right). (A) Representative hematoxylin–eosin (H&E) stained sections and immunohistochemical analyses show a characteristic Burkitt lymphoma phenotype with strong expression of CD20, CD10, BCL6, and c‐MYC, absence of BCL2 expression, and a Ki‐67 proliferation index approaching 100% in both specimens (original magnification × 400; scale bar = 60 µm). (B) Immunoglobulin heavy‐chain gene rearrangement analysis demonstrates distinct clonal patterns between the two samples, with clonal bands detected in framework regions FR1 and FR2 in the 2015 specimen and a different clonal rearrangement localized to FR3 in the 2023 specimen, supporting the presence of two molecularly distinct disease occurrences.

Despite these shared morphological and immunophenotypic features, clonality analysis revealed distinct IGH rearrangement patterns between the two specimens. The 2015 lymphoma showed clonal rearrangements involving FR1 and FR2 regions, whereas the 2023 specimen demonstrated a different clonal band restricted to the FR3 region, indicating a lack of clonal identity. These findings are illustrated in Figure 1.

Next‐generation sequencing further supported this distinction, identifying pathogenic mutations in *BRAF* (p.G466V) and *PTEN* (p.G127R) exclusively in the tumor sample obtained in 2023. These variants were absent in the 2015 lymphoma specimen as well as in tumor‐free control tissue. Collectively, these molecular findings support the interpretation that the 2023 disease represents a biologically distinct BL rather than a true late relapse of the original malignancy. The corresponding results are displayed in Table 1.

**TABLE 1:** Comparative NGS analysis of Burkitt lymphoma (2015 vs. 2023) and tumor‐free bone marrow: Identification of constitutive polymorphisms across all specimens and specific BRAF and PTEN gain‐of‐function alterations unique to the 2023 tumor tissue.

Gene	Mut. effect^a^	HGVS c.	HGVS p.	2015 (1) Tumor	2023 (2) Tumor	2023 Normal tissue
				VAF (coverage)		
**BRAF**	**5**	**c.1397G>T**	**p.Gly466Val**	—	**40% (1746)**	—
CSF1R	1	c.*35_*36delinsTC	—	91% (1961)	92% (2522)	95% (2087)
EGFR	1	c.2361G>A	p.Gln787=	98% (1847)	100% (2434)	100% (2479)
FGFR3	1	c.1953G>A	p.Thr651=	100% (2767)	100% (3681)	100% (2865)
FLT3	2	c.1310‐3T>C	—	50% (6103)	48% (3671)	49% (4187)
KDR	3	c.2618G>A	p.Gly873Glu	42% (2387)	—	—
MET	3	c.2975C>T	p.Thr992Ile	51% (4434)	50% (2736)	60% (3733)
PDGFRA	1	c.1701A>G	p.Pro567=	99% (3405)	100% (3481)	100% (4466)
PIK3CA	1	c.352+40A>G	—	52% (3001)	49% (1267)	53% (2154)
	1	c.1173A>G	p.Ile391Met	55% (3454)	54% (1622)	55% (2674)
	3	c.2038G>C	p.Val680Leu	40% (3513)	49% (2992)	48% (4103)
	2	c.2155C>G	p.Leu719Val	40% (3311)	48% (2902)	47% (3994)
**PTEN**	**5**	**c.379G>A**	**p.Gly127Arg**	—	**77% (2783)**	—
RET	1	c.2307G>T	p.Leu769=	99% (4606)	100% (5150)	100% (4225)
SMAD4	—	c.354G>A	p.Ala118=	24% (2734)	50% (2521)	50% (3944)
TP53	1	c.215C>G	p.Pro72Arg	100% (1413)	100% (2797)	100% (3098)

*Note*: Bold font indicates pathogenic or likely pathogenic variants identified exclusively in the 2023 tumor specimen.

Abbreviations: HGVS c., Human Genome Variation Society coding DNA; HGVS p., Human Genome Variation Society protein; Mut. Effect, mutation effect; VAF, variant allele frequency.

^a^ Variant pathogenicity was assessed using the five‐tier ACMG/AMP classification system (Classes 1–5), ranging from Class 1 “benign”, 2 “likely benign”, 3 “uncertain significance”, 4 “likely pathogenic” to Class 5 “pathogenic” [19].

At the time of the secondary diagnosis, there was no evidence of central nervous system or bone marrow involvement.

He was initially treated with the salvage regimen of DA‐EPOCH‐R, combined with intrathecal methotrexate. He received 6 cycles, and although there was a transient clinical response, disease progression was documented by a positron emission tomography and computed tomography (PET‐CT) scan in October 2023, which revealed new lesions in the liver, spleen, and kidneys.

Although these FDG‐avid lesions appeared small in volume, biopsy confirmed active BL, underscoring that even minimal PET‐positive disease in this setting can represent aggressive and clinically meaningful relapse.

A second salvage regimen was initiated, including rituximab, ifosfamide, carboplatin, and etoposide (R‐ICE) in combination with intrathecal triple therapy. A follow‐up PET‐CT scan in December 2023 showed partial metabolic remission in the liver and spleen. However, new hypermetabolic lymph nodes appeared in the hepatic hilum, peripancreatic region, and retroperitoneal. Based on these findings, disease progression was assumed, and no confirmatory biopsy was performed at that time.

Worsening lymphopenia rendered the patient ineligible for chimeric antigen receptor T‐cell therapy within the ZUMA‐25 clinical trial.

Glofitamab therapy was initiated in December 2023 as an off‐label treatment due to the lack of other meaningful treatment options. Following step‐up dosing during the first cycle, glofitamab was administered at a fixed dose of 30 mg intravenously every three weeks for 12 cycles in total. At the initiation of glofitamab therapy, the patient's LDH level was 260 U/L, consistent with a moderate tumor burden and relevant for contextualizing the risk of CRS; however, no cytokine release syndrome (CRS), immune effector cell‐associated neurotoxicity, or other unwanted reactions were reported. Infectious monitoring included regular HIV viral load testing, which remained undetectable throughout treatment, as well as serial Epstein‐Barr Virus and Cytomegalovirus PCR testing, which showed no evidence of viral reactivation. Prophylaxis with co‐trimoxazole and valaciclovir was continued during therapy. Immunoglobulin levels were monitored and remained stable, and immunoglobulin replacement was not required. No clinically significant infectious complications occurred under glofitamab.

Therapeutic response was monitored with both imaging and histopathological assessments.

A PET‐CT scan in February 2024, after three cycles, displayed an almost complete metabolic remission from all lesions, apart from perihepatic and perisplenic lymph nodes, where an increase in FDG avidity was documented.

A laparoscopic lymph node biopsy of the hepatic hilum, however, showed no residual malignant infiltration, and treatment was continued, as the biopsy revealed a granulomatous, non‐necrotizing, but advanced sclerosing lymphadenitis.

A PET‐CT scan in July 2024 confirmed stable FDG uptake, consistent with partial metabolic remission (The Lymph nodes were interpreted as sarcoid‐like lesions). The lymph nodes in the hepatoduodenal ligament measured up to a maximum of 5 mm at this point.

In total, 12 cycles of glofitamab were completed (until 08/2024). Subsequent imaging showed complete remission in October 2024 and again in March 2025, with the latest scan conducted seven months post‐treatment. The patient remained in good clinical condition at the time of last evaluation in December 2025 with no evidence of relapse.

**FIGURE 2:**
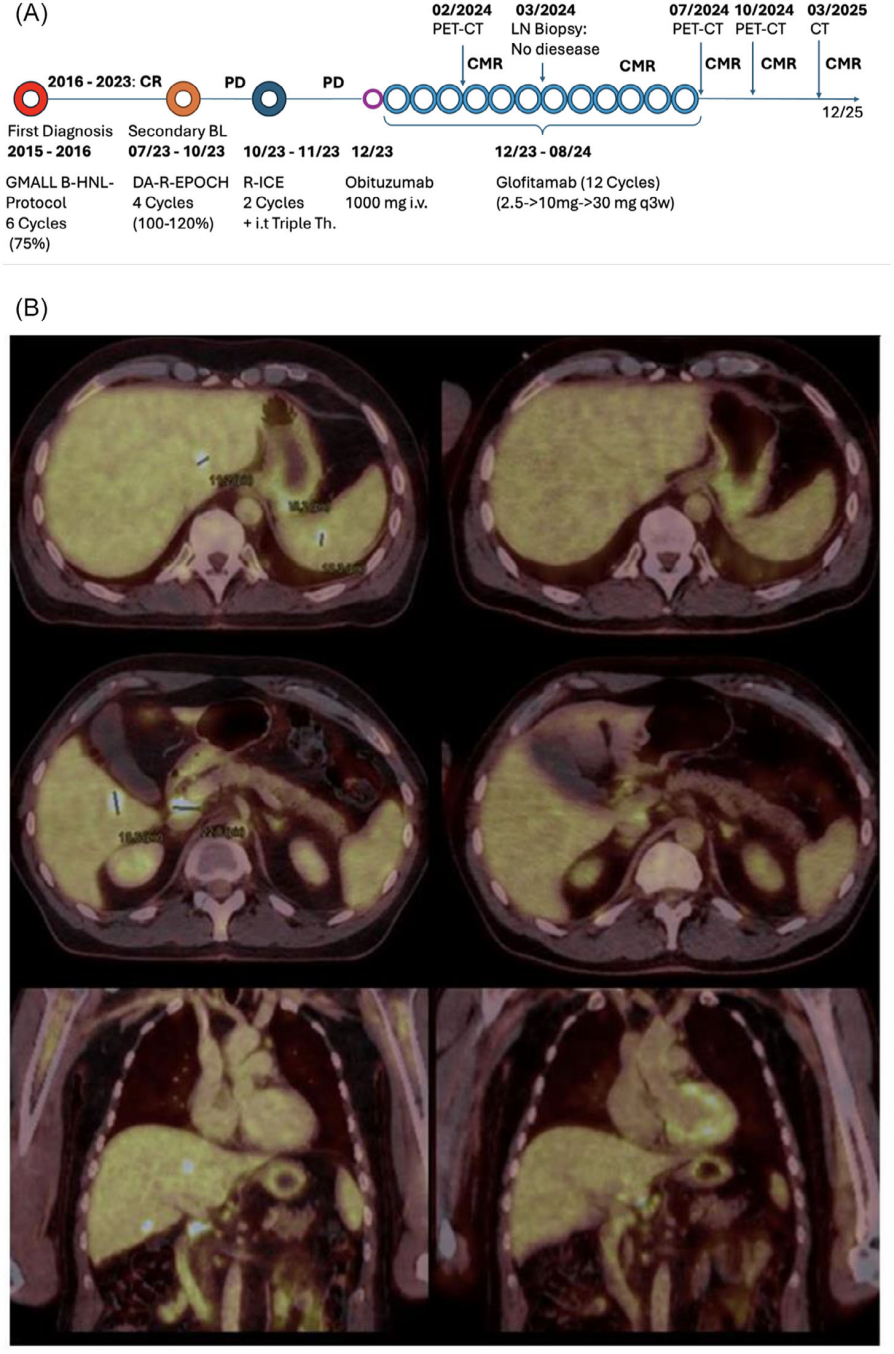
Timeline of treatment and clinical course with positron emission tomography and computed tomography (PET‐CT) comparison. (A) Timeline of treatment and clinical disease course from initial diagnosis to remission. (B) PET‐CT comparison between October 2023 and October 2024 demonstrating complete metabolic response to glofitamab therapy.

The overview of the timeline of treatment and clinical disease course from initial diagnosis to remission, as well as the PET‐CT comparison between 2023 and 2024, is depicted in Figure 2.

## 4 Discussion

This case highlights the clinical efficacy of glofitamab in a heavily pretreated, 60‐year‐old patient with refractory BL. The treatment was well tolerated, without complications.

Glofitamab was administered off‐label in the absence of viable standard treatment options, and it was chosen based on its approved and effective use in diffuse large B‐cell lymphoma [15, 16, 17].

The distinction between late relapse and second primary BL is clinically relevant. While morphology and immunophenotype alone are insufficient to discriminate between these entities, the integration of clonality testing and next‐generation sequencing provided decisive evidence. The absence of shared IGH rearrangements and the presence of unique pathogenic mutations in the 2023 specimen strongly argue against clonal relatedness and support classification as a second primary BL. These findings support the interpretation of a biologically distinct BL arising years after initial remission.

Importantly, this molecular distinction does not diminish the clinical relevance of the present case. Regardless of whether the 2023 disease represents relapse or a second primary malignancy, the patient exhibited aggressive, treatment‐refractory BL with limited standard therapeutic options.

The discordance between persistent FDG uptake on PET‐CT imaging and the absence of malignant cells on histopathological examination raises the possibility that the PET‐positive lesions observed after R‐ICE may, at least in part, have reflected inflammatory granulomatous activity rather than persistent lymphoma. Although this scenario is considered unlikely in the context of the overall clinical course.

The durable response achieved with time‐limited glofitamab therapy, therefore, highlights the potential role of CD20xCD3 bispecific antibodies as an effective salvage strategy in this high‐risk clinical setting or immunocompromised patients.

This case supports existing data indicating its effectiveness in patients with HIV‐positive‐associated B‐cell lymphoma, a population often excluded from clinical trials involving immunotherapies [15, 20].

This case holds relevance for clinical practice, especially considering the most recent data published from the EBMT registry, showing the modest benefit of auto‐ and allo‐HSCT in patients with BL [7].

A review of the current literature on bispecific antibodies in relapsed BL, as reported in Table 2, reveals only five reported cases in which this type of therapy was successfully used, and in three of these cases, an allogenic transplantation was planned or performed after the successful bridging therapy [14, 18].

**TABLE 2:** Glofitamab in relapsed/refractory Burkitt lymphoma.

Reference	Diagnosis	No. of patients	Prior therapies	Cycles glofitamab	Assessment method	Best response	Further therapy
NEJM 2025 (https://doi.org/10.1056/NEJMc2501018) [18]	Refractory Burkitt lymphoma (HIV‐status unknown)	3	Patient 1: Five prior lines incl. DA‐EPOCH‐R, R‐GDP, radiotherapy Patient 2: Three prior lines incl. R‐CODOXM‐R, R‐ESHAP, radiotherapy Patient 3: One prior line R‐hyper‐CVAD and R‐MA	2–5 cycles of glofitamab + polatuzumab vedotin	PET‐CT and biopsy	Two CMR, one PD (after CAR‐T failure)	Two allo‐SCT, one resumed glofitamab + planned allo‐SCT
T&F, Leuk lymphoma 2024 [14]	Relapsed Burkitt lymphoma (HIV‐negative)	1	Three prior lines: R‐EPOCH, high‐dose MTX, auto‐HSCT	Six cycles of glofitamab	PET‐CT and clinical exam	CR	Observation (ongoing remission)
Current case	Refractory Burkitt lymphoma (HIV‐positive)	1	Three prior lines: GMALL B‐HNL, DA‐R‐EPOCH, R‐ICE	Twelve cycles of glofitamab	PET‐CT and biopsy	CMR confirmed by biopsy	Observation (ongoing remission)

The patient's sustained remission, even though no consolidating HSCT was performed, the absence of serious adverse events, and overall good clinical tolerance suggest that bispecific antibodies may not only serve as a valuable bridging strategy but also as an alternative to cellular therapies in high‐risk or immunocompromised patients.

## 5 Conclusion

This case underscores the potential of glofitamab as a stand‐alone, time‐limited therapeutic strategy, rather than a bridging therapy, especially in patients ineligible for consolidation with HSCT due to refractory disease or comorbidities.

## Author Contributions

Elisa Bozzotto and Thomas Melchardt were responsible for manuscript drafting and data integration. Conceptual oversight of the manuscript was provided by Theresa Magnes, Alexander Egle, Dominik Kiem, and Daniel Neureiter, who conducted the histopathological evaluation of the Burkitt lymphoma specimens and generated the associated table and figure. Barbara Zellinger performed and interpreted the molecular analyses. Arno Beer contributed clinical expertise related to infectiology and HIV management. All authors participated in manuscript revision, critically reviewed the content, and approved the final submitted version.

## Conflicts of Interest

Alexander Egle received Honoraria for educational talks and travel assistance from Roche. Arno Beer has received travel, accommodation, and expense support from AstraZeneca, MSD, ViiV, and GSK, and speaker and/or advisory board fees from Gilead. Dominik Kiem has received travel, accommodation, and expense support from Roche. All other authors declare no conflicts of interest.

## Funding

The authors have nothing to report.

## Ethics Statement

The patient was included in the Lymphoma Registry of the Austrian Group for Medical Tumor Therapy (AGMT) Study Group, and the Registry was approved by the institutional ethics committee (EK N:1169/2023). Written informed consent was obtained from the patient for inclusion in the registry.

## Consent

Written informed consent was obtained from the patient for inclusion in the AGMT Lymphoma Registry and for the use of anonymized clinical data for scientific analyses and publication.

## Data Availability

The data that support the findings of this study are available from the corresponding author upon reasonable request.
